# Pyorrhea Alveolaris—The Result of Disease

**Published:** 1884-02

**Authors:** G. A. Mills


### THE DENTAL REGISTER.

VOL. XXXVIII.] FEBRUARY, 1884. [No. 2.

### COMMUNICATIONS.

### Pyorrhea Alveolaris—the Result of Disease.

### ITS ORIGIN, ETIOLOGY, AND TREATMENT.

The subject of this paper was selected in response to an invitation received on the 23d of June last, from Dr. Willoughby Miller, of Berlin, Prussia, requesting me to meet with the society of American dentists, at their annual meeting, to be held August 7th, at Cologne, Germany, and to give them my views and experience in connection with the special treatment of Pyorrhea Alveolaris; Dr. Miller saying in his letter that "it is a subject about which we know almost nothing over here. The same statement would be true, I think, with few exceptions, the world over."

Having had a lifelong desire to visit Europe, I accepted the invitation by saying I would prepare a paper, and could I possibly come I would do so. I was not able to go, and, as I had not been able to complete the paper, I concluded that I would defer the whole matter, in the hope that the time would yet come when I would have the pleasure of meeting the dentists of Europe and seeing the country. Therefore, should my thoughts upon the subject I have selected prove in any sense helpful to this body, I shall be gratified.

This subject of the disease of the gums, loosened teeth, etc., has been written upon under a variety of headings—Riggs' disease, pericementitis, phagedemic pericementi, catarrh of the gums, premature wasting of the alveolar process, infectious alveolitis, etc. As Dr. Miller suggested pyorrhea alveolaris, I thought best to give my views regarding it as the *result* of a disease, rather than as a disease of itself. This same view I would apply to all the lesions taking place in connection with the human organism. Possibly I may not succeed in making my views plain to the so-called *practical* portion of our callings; nevertheless I will make the effort, trusting that I may excite more mental action and help to create a thirst for a better understanding of occult things. Should I undertake to give my thought regarding the subject of dental caries, that is occupying so much attention at the present time, I should proceed on the same basis as I propose to with the subject of this paper.

I view the days in which we are living as full of hope, encouragement, and helpfulness to those who are devoting themselves to the amelioration of human suffering. I am not at all in sympathy with the feeling I hear much expressed of late, and by those who have been long at their work, that ours is "but a bread and butter calling; if I were to live my life over again I would take up some other profession. What is a dentist?" Admitting that we are not all that we should be, and that we hope ultimately to be, what are such discontented men doing, and what have they done to make the profession better by becoming one of us? When a man, on leaving a place in which he has sojourned for a time, remarks, "I have had enough of that town," people say, and I think with reason, that he has evidently allowed something to occur that is not altogether to his credit; and so I think the expressions of dissatisfaction to which I have alluded suggest a similar reflection upon the professional careers of those who make them. Some of the bright members of our profession—some who have written books even—have gone out of their sphere of earthly activity with that sad lamentation on their lips, "nothing but a bread and butter calling." Grand is the man who struggles through all the discomforts, jealousies, envy, and privations which are met in the pursuit of his chosen calling, and at the end lays aside his armor with his manhood untarnished and the garland of victory illuminating his face, indicating hope for a better future.

But, gentlemen, I have not come to you with the thought of throwing out an intimation of the decline of our calling; there is no more indication of it than there is with respect to any of the other callings of life. As in the physical development the continuous processes of waste and repair are busily at work, so with us new energies are coming in, with a higher potentiality for evolution than the past has ever known. The avenues of knowledge are being abbreviated; the truth is no longer sequestered in convents of mystery with padlock and chain to limit its distribution; the day is past for the knowledge of the times to be condemned or rendered orthodox by the votes of any body of men. So let us rejoice and take courage, and give our allegiance, and our highest and best efforts to *all* that is helpful, wherever found and by whomsoever claimed, esteeming it divine because it gives life, hope, and relief from suffering; and away with the clamor of avarice for originality, which so lessens our ability to afford the immediate aid which the human race needs and is waiting to receive.

Those of you who are at all familiar with my writings have doubtless discovered, to some extent, the enthusiasm that enters into my purposes, the depth and sincerity of my convictions, and that my understandings have no ground to occupy but the advance, which they hold with a tinge of the aggressive spirit.

My attention to the subject of this paper was first enlisted through the courtesy of Dr. Searle, of Springfield, Mass. Chancing in 1868 to stop over there on my return to New York from Boston, where I had been filling an engagement of clinical teaching in the Boston Dental College, I was invited by Dr. Searle to visit Hartford, Conn., and witness an operation by Dr. John Riggs in the mouth of a patient whom he was to take there for Dr. Riggs' special method of treatment. At that time clinics were very popular, although they are now falling somewhat into desuetude with us, for various reasons which I will not attempt to detail here. The operation was made on the lower front teeth, which were quite loose, there being a considerable destruction of both the hard and the soft tissues. The instruments and their use were new and strange to me; I listened with no little interest to Dr. Riggs' discoursing as he operated, and his intelligent presentation of the history and outcome of his prolonged observations in the direction he was pursuing; this made a lasting impression upon my mind, which has become more indelible as I have endeavored to educate myself in the same field of labor. On my return to Brooklyn I rehearsed my observations to the Brooklyn Dental Society, and suggested the idea of inviting Dr. Riggs to present his views to us in connection with a clinical demonstration. In due time this was accomplished. At that time we had a dental infirmary connected with the society, supposed to be the only one in existence outside of a school. It was inaugurated by the society and kept in active life for something over five years, receiving an annual appropriation from the city of $1,500. This institution has ceased to exist, for reasons which need not be stated here. Dr. Riggs spent a number of days with us, treated several cases, and presented others which were in course of treatment by him ; and in these partly treated cases the contrast of health and disease was so decidedly shown as to establish beyond question the value of his methods of treatment. I am particularizing here with a special purpose, which is to show how hard it is to educate the dull mind and the unenlightened perception up to an appreciation of the value of an important feature of practice, and how slowly recognition follows its introduction. Farther on I will refer to this again, in connection with what has been recently said on this subject. The importance of this subject is beginning to be acknowledged, and the honor accorded—to whom? To those who have been active and diligent in agitating it? Oh, no! "We always thought so!"

As I have said, my deep interest in this subject grew and became more potential with every opportunity that presented itself to me of acquainting myself with Dr. Riggs' views and practice. Good fortune, as it now seems, provided and extended opportunity for me to familiarize myself with his actual office practice in Hartford, which I assiduously improved by standing over his chair, from day to day, as often as I could find time to do so. As a result of this I saw a fuller confirmation, by a larger number of cases, of the great value of his treatment. In the meantime I submitted to the examination of my mouth by Dr. Riggs, which had been in the hands of prominent practitioners who were reputed to be among the best in the profession. In my mouth I was shown a discharge of pus about the bicuspids and molars. Up to to this time I had leaned to the current idea that Dr. Riggs' operations were very severe, but my confidence had become so strengthened by the results I had witnessed--and it is confidence that carries us successfully through more than half the battles of life—that I decided to know for myself, and arranged to have my mouth treated. It was done. That was ten years ago last June. The results were all that could be desired, and I have had them revised from year to year. This experience at the hands of Dr. Riggs reduced my convictions to personal knowledge, and from that time I have been an undeviating believer and follower of his practice. And I wish to note this with an emphasis. I have repeatedly called attention, in my writings and reported sayings, to the *Riggs' feature* of the disease, which is the necrosed edge of the process, as I have called it, or the non-nutrient condition which Dr. Atkinson has so often defined. Dr. Boedecker has just made an effort to criticize me in this connection, as you will soon see in the published proceedings of the First District Society of New York, and I deem this a good place to answer him. He falls into the error of making a distinction *without a difference*, as between "mal" nutrition and a necrosed or non-nutrient condition. And I wish to note another point just here, as an indication of the peculiar development of understanding that is now occasionally noticed. It is in connection with the presentation of a case of office practice by Dr. Boedecker, in which loosened teeth had been made firm. This will be embodied in the same report I have referred to above. In replying, Dr. Atkinson said: "It is a matter of no little astonishment to me that such possibilities as have been detailed by Dr. Boedecker should be spoken of now as something *new*, for it is a well known fact that I have been reporting for the last twenty years, cases that have demonstrated the restoration of tissues, both soft and hard." Dr. Atkinson *has* proved beyond a denial, in his practice, that these tissues can be, as a practice, reproduced. I say *as a practice;* for all we have to do is to acquire a knowledge of principles, which form the roadway on which all progress may easily glide. And we must have a knowledge of these principles before we can, with anything like certainty or uniformity, establish by them a helpful and successful practice.

The reproduction of tissues about the apex of the root of a tooth that have been destroyed by an abscess, is no different from the process of reproduction of the same tissues by the *side* of the root or about it, when they have been removed, as in the case of prolonged pericementitis. In the first case, if the abscess is chronic, having a fistula, and is to be aborted, we have but to take a suitable burr in a dental engine and delicately follow the indicated track of the disturbance to the seat of the disarrangement, and by the *trained sense of touch* we are able to readily cut away all the non-nutrient material down to that which is normally nourished; then, in most cases, simply leave it in the clot and the parts will return to health. That this is but simple surgery all educated minds will admit. Now, is this new? I answer, no; but it is old knowledge with a new understanding and application. And here I wish to emphasize an established truth, which is that the feature of this disorder—call it Riggs' disease, because first observed and explained by him, or call it a non-nutrient condition of the process if you please—the character of the disorder is the same as in the abscess; and the same procedure and methods of treatment that prove most efficient in the case of the abscess will also prove efficacious and bring about the desired results when applied to the disease under consideration. It is but *simple surgery* applied to this department of practice, it is only a new understanding applied to the treatment of a long known and terribly destructive malady, and one which, in my opinion, causes the loss of more teeth than all others combined. With present knowledge we *ought* to be able to produce, *as a practice,* a state of health in the oral cavity that will do as much, if not more, to answer the perplexed question of dental caries as all else that has been brought to bear upon it yet.

And at this point in my paper I will call attention to a feature that is not, that I have any indication of, being much considered; certainly not in any proportion to its importance. It is the incipient stage of the disorder, as manifested in a very large majority of mouths. In the early part of my writings I have referred to the desirability of making this subject one of first interest, observation, and importance, in the daily practice; for when I notice the delineation of cases such as I have referred to, I cannot help asking: if the process of destruction which causes the loosening of teeth arouses the attention, why does not the general state of the others also enlist the same interest? For I challenge the presentation of a case of absolute freedom from a progressive expression of the same state of things. If the *few* are so far advanced in the process of disarrangement, you will certainly find that the others need the attention of a trained intelligence; for it is largely by prevention that the progress of the disorder must be checked. Further on I will refer again to this point.

I will now come to the more direct consideration of this subject which is expressed in the title head of the paper, "Pyorrhea Alveolaris; the Result of Disease."

By pyorrhea we mean a flowing. In a normal condition of the tissues we have a flow of what are termed the juices of the flesh, while the process of endosmosis and exosmosis, or inward and outward penetration, is continually going on. This process is under the control of what are called the organs of secretion and excretion. These are also in connection with and acted upon by the other auxiliary organs of the body proper, and play an important part in keeping at work the functional activities of the constitution; and while all this goes on harmoniously we have, as the legitimate and natural result, health, ease. The type formed by the fullest expression of nature's laws means only health, or a sound, perfect, and satisfied condition. That this condition is the one which nature designs I do not for a moment doubt; that we have yet seen any of the full type conditions I do doubt. The saying that "it doth not yet appear what we shall be," is, I am certain, as applicable to all the relations of the processes of creation as it is to the state of man in a future world; but when that condition of perfect health shall be obtained, the human race will have reached "a new heaven and a new earth," and the right-livers only shall dwell therein; for perfect health and sin are, in my opinion, wholly incompatible. I do believe that the time will come when these bodies of ours, now so imperfect, will be free from the imperfections of perverted nutrition; and just in the degree in which we obey the laws of our being, will we advance toward that happy age when all men shall be well born and have perfect health as an inheritance, and when the processes of generation will have become so complete that there will be no need of *regeneration*. This thought is not lightly uttered, and I shall be glad if I can so vividly impress it upon your minds, with its important bearing upon our professional work, that you will all be enabled to look forward more hopefully to a better future, to a more healthful and intelligent expression of practice, and thus be inspired to endure the toil and privation by which only it can be attained.

By this seeming digression I have directed the thought to a state of health, which is *ease*, and your imaginations will readily picture an oral cavity set with priceless pearls as white as the sheep from the washing, a beautiful glow of blushing life in the soft tissues caressing their necks, the virgin dew of health exuding from their surfaces, and giving out a sweet odor that rises toward heaven as incense of praise.

This, I acknowledge, is the ideal; but I know of no better maxim to preface each day's purpose with, than that of the progressive thought of St. Paul: "I count not myself as having attained;" but I will leave nothing undone that I may obtain the truth.

Doubtless you will expect something to indicate my thought regarding the origin of disease, and I will try to give it by making an exhibition of conditions opposite to those of health, and which are *dis*-ease.

Turning to an examination of a typical state of things that we commonly see in practice, we will present a fine set of teeth, nicely arranged, a good facial appearance, with scarcely any defect; we notice, when in conversation, a peculiar odor, of a sickish, fœtid character. On separating the lips, to a discriminating mind with an educated eye, lines of unhealth are at once noticeable on the labial surfaces of the front superior teeth; the gum tissue pale, loose, and anæmic in appearance. With a delicate probe you will find the tissue detached to varying distances of from a quarter to half an inch perhaps; a perceptible flow of pus will follow by pressure; a roughened edge of the process is easily detected—note *this*—to the *trained sense* of touch. Often on the labial surface of the gum there will be seen a pimpled appearance. Now, as a general observation, these phases will not be taken as expressions of *dis*-ease. Again, elevate the head a little and take a look, with a mouth-glass, at the palatal portions of the same teeth, and notice the decided difference of color in the tissue —a blue, or purple stained and shiny appearance. Put the ball of the finger upon it and it will have a patulous feel. Probe, and you will find extensive waste of both soft and hard tissues, forming a deep pocket, holding not a little deteriorated, broken down tissue, formed of pus and debris. Both of these expressions are the result of *dis*-ease. It is pyorrhea alveolaris—pus discharging sockets—*not a disease*, but the *effects* of one. I do not consider it necessary to enter upon a lengthened detail of the various phases of the disorder in question, and yet if I should give my frequent experience it would indicate that the time is not past for giving line upon line and precept upon precept, until a more intelligent perception prevails in regard to the vivid manifestations of unhealth that are now so disregarded among the mass of practitioners; and I do not say this from any ill-thought. We must admit the fact that our branch of labor is yet, in a comparative sense, in its infancy, and our prospect for higher attainments is as much a *fact* as the former. No man has a greater admiration and hope for the future of our special field of usefulness in the amelioration of human suffering than myself, and it is because of this that I am kept alive in the purpose of doing what my heart, head, and hands find to do to encourage the weakest —if earnest—to secure a part in the same.

You will see by this time on what my thoughts are based, in support of theories regarding the origin of disease. And I will take your minds a little further into the labyrinths of theorizing, for it is no longer considered unsacred to *try* to find out the workings of the Creator. And just here I must refer to the exceedingly interesting and profitable results of Prof. Carl Heitzmann's labors.

We have in his book upon the morphology of the tissues, published now both in English and German, a revealment to the mental and ocular vision that certainly leads us into not only advanced fields of understanding, but of an easier cultivation, vastly more prolific in quantity and with a potency of increased power for growth. I can see no possibility of an exact science coming to our aid as a helpmeet in the leading of mental action but to consider all questions from a portraiture of the result of law. The artist by his interblending with colors and forms only succeeds in bringing satisfaction towards his ideal but in proportion to his achievement of harmony. It is then only that Pygmalion stands before his inanimate statue Galatea and agonizes to the gods the full desire of his heart (as yet unuttered); but to him in this form, though of cold, lifeless marble, he sees the harmony of his aspirations so complete that he breathes spontaneously out his own soul to the source of life, vividly hoping that the zephyrs will be moved to return their living breath into the nostrils of this virgin form that it shall become a living soul. We have now but to follow the analogy and apply it to other fields of effort to secure ultimately the joyous expressions of healthy action. Now I am fully aware that there is a disposition afloat to regard such views as I have given expression to as "sentimental," "all in the air," etc. Yet, we cannot ignore the fact that there is foresight as distinct as present or hindsight. All these are actively engaged in the investigations of the day, as much as, if not more than, what we denominate the more tangible operations. Mind is always a precedent to matter; the first objective, the latter subjective; and it can be only by these auxiliaries that we can delve into and explore the unseen. We do *now know* some of the once supposed *unknowable* things, and we *will* yet know more of primary causes. The time will come when we will be bringing out to the understanding a knowledge of typology and cunentology, which will contribute an understanding to morphology that we are not yet in possession of. In that mysterious little ameba, unseen but by the higher power of the microscope, is a knowledge, though yet only able to project its pseudophobic movements, *will* walk, yea, it will run, like the swift courier, to meet the increasing demands of a diviner order of thought. Truly we do yet know but in part, but as the higher living shall become more generated in the world a potency will make itself felt that will totally eclipse all our best possibilities of to-day. Alas, how unmeaning a word is that subtle expression, potency, to the mass of minds! As Oscar Wilde says of the American girl, "she is like an oasis in the desert of common sense." But let us rest in hope until the prophecy shall be fulfilled that "all deserts shall yet blossom as the rose."

I have considered in my own mind the value of a resume of some of the various views that have been published of late by Drs. Majitot, Weitzel, and Black. If these several writers had given us their reasons for the thought they have expressed, it would have facilitated matters. Majitot is published as saying that these expressions of disturbance about the gums and sockets of the teeth are "always premonitory symptoms of diabetes." Seeing these statements, I sought to know the facts, and applied to Dr. Davenport, of Paris, to procure me the writings of Dr. Majitot, such as should embody the views published by him (as reported). To the courtesy of Dr. Davenport I am indebted for the receipt of a paper. Subject: "*Tumuers du perioste dentaire, et sur l'osteo-periostite alveolare dentaire.*" The date of this published paper is 1873. On page 89, under the head of diagnosis, he includes physical irregularities, viz., diabetes, cephalic congestions, hemorrhoids, suppressions of menses, etc. In connection with these views I have sought to learn how far they could be sustained. The first opportunity that came into my hands for observation in this line of thought was a lady patient last autumn. This case had been noticed by the practitioner who had had charge of the oral cavity in connection with general dentistry, filling, etc., but so far as the health of the mouth was concerned nothing had been done more than to say it was a serious malady, and involving the probable loss of the teeth. The age of the patient was about twenty years. The destructive effects were manifested by a marked waste of the attachments, scarcely any external deposits, a copious discharge from the deep-seated pockets, and some scattered nodules on the roots of several teeth; the patient was of a bilious temperament, with a touch of the melancholic expression, and a decided anæmic presentation generally noticed. Teeth finely formed. I consulted the father, told him the views oi Majitot, as reported, and asked his co-operation in procuring a microscopical diagnosis. He readily consented, and took a portion of urine to Dr. Heitzmann, with a letter of introduction to him. In his written statement, which I find is mislaid, the only point of interest he traces is a slight trace of albumen, more associated with leucorrhoea, which, he adds, is quite common to females. This being so marked a case, I felt an unusual interest to see what could be traced in the line of physical irregularities. [I have felt for a long time that a phase of the disorder in question, as manifested in the case mentioned, and two others very similar, both females, and apparently about the same age, although of quite different temperaments, were symptomatic of irregularities of the generative and urinary organs particularly.] I do not think, however, that any general application can be made of these points to the disorder we are considering, while I shall hold that the general expression is the result of perverted functional action, or neurosis of that branch of nerves that preside over the process of assimilation; and yet I see no other conclusion to arrive at, as a general or a first cause, if I may so express it, but depleted nerve force. The nervous system holds the supreme control of all organic function, and in proportion to its depleted power functional activity is lessened. I see no ground whatever for entertaining the doctrine of "infectious alveolitis;" certainly in the sense that the adjective infectious is used—as a poisoning, or a septic action. This only can be possible under very adverse circumstances, forming what I would term a susceptibility to septic poisoning; and such conditions could only be matured associated with this disorder but by a very general systemic disarrangement, usually termed "general debility." I know it will be argued that because the disturbance is progressive and going from tooth to tooth it is necessarily septic. Dr. Black, in his article upon phagedemic pericementi, says he leans to Dr. Weitzel's views. Of course he must if he views the disturbance as a syphilitic ulcer, the Greek meaning of his term. Now here are three writers giving their think-so, but not the whys. Dr. Davenport writes me that Majitot has another paper that is to be published, in which he is to give his reasons for his views. It has been said by some that have talked in a general way of this subject, that they did not accept the theory of "nerve degeneracy" because the cases they had noticed showed no signs of it. What is the standard they judge by I ask? A patient does not necessarily need to be a marked case of neuresthenia and all its concomitant associations, in order to be classed under this head. The definitions of the mass of men who write and talk, when pushed to the absolute, do not stand. It is not an uncommon occurrence for patients, when their health is referred to, to say, often quite emphatically, that they never had a sick day, etc. That may be in the sense they mean, but there is a vast amount of unhealth that is not put to bed and called sick. A liver, heart, lung, kidney, etc., can be far from performing its normal functions, and the patient be about the general activities of daily life. The sudden taking off, which is frequent in these days, and is the occasion of so much surprise by the lay portion of the public particularly, and which finds expression in this wise: "Why they were the pictures of health;"--the possibilities of such a sudden termination have been accumulating for months, and doubtless years, in many cases. At last nervous degeneracy yielded to the stronger force that had accumulated, and the tissues gave out at a given point, and the spirit rose out of its earthly or material habital to a more genial clime in which to exercise its better capabilities. We can study causes only by noticing and understanding effects; action and reaction must be made understandable by intelligent and unprejudiced investigations. Disarrangement of the kidneys gives a result expressed by stone in the bladder. We also find it in gouty and rheumatic disturbances indicated by hypertrophied deposits at the joints, also as in gall-stones, and salivary concretions manifested externally upon the teeth--a residuum of organized mineral substance in so-called dental caries. And lastly the debris of the inflammation of the pericementum.

In speaking of the treatment, while I cannot go over in detail the modus operandi in the use of instruments, medicinal aids, etc., yet there is vastly more to be taken into consideration than the mere statement. I might as well in the start make this emphatic declaration, and I do it in part to answer the many inquiries that come to me by letter and otherwise. It may seem to some as being opinionated; if so I cannot help it. I do not propose to make as many mistakes as I have by doing what I meant for kindness, but which have proved often the reverse. A large experience has taught me, if it has no one else, that it is far more helpful to go slow. If a young man comes to me and says he thinks of becoming a dentist, I am in the habit of entering into an interrogatory conversation for the purpose of an examination, and, if possible, to get his status of thought regarding the calling he wishes to pursue, and before I get through I form my judgment of his possibilities. You may guess what the average presentation is. So, also, I find in connection with this subject about as superficial a thought with the mass of inquirers. And yet "they all do it." Now, until I see vastly more evidence of the knowledge that is *claimed* put into practice, and find a better understanding evinced by the great mass of patients I come in contact with, I shall persist in continuing to manifest the same earnest and persistent purpose that I have. As I have said, I say again, I see a mission in this field of neglected harvest, white, and waiting for the laborers; and they are few, very few, and will be until there is a steady purpose to be put into tuition and receive a demonstrated, practical training. That there is an opportunity for such teaching in any school I am not aware of. Why do I say this? I am compelled to do so, for the students of all the schools are my informers. I do not mean that the subject is not made mention of, but I do mean that a systematic course of teaching, such as I have referred to above, has not been put into practice, so that *all* the students shall leave that school with the impression that it is of the greatest importance, and that it should be the *first* step in *all* practice, with the *youth* and the *adult*. To simply tell the student this does not suffice; he must be *clinically* and systematically, as with other branches, instructed throughout the college course. I ask every fair-minded practitioner if he sees evidence enough to convince him that this is being done. I will give one example, which I admit is an extreme one. A fresh graduate called on me last spring and said he wished I would give him my method of treating diseased "gooms." As I am giving samples it may serve as a little diversion to give a few more. A case was presented to me by a dentist for my opinion of his own mouth. He said *he believed* it was the result of hay fever. Another by a graduate in medicine who brought a patient for filling. The mouth was in a disgusting condition; the patient a lady, and his sweetheart. I told the M. D. the first need of the mouth, and made an exhibition of the unhealth, gave my views, etc. He replied that he could give a prescription for that, and insisted upon doing it. This was five years ago. I have been told within two months that the patient is in a serious condition of health; and this mouth has never received the needed treatment, and advised still another. A mother refused to let me *scrape* her son's teeth, and applied to one of my neighbors and had him *agree* not to scrape the teeth. He did. This dentist took a fee of $70 for filling, and left the mouth with the destructive agencies remaining. Yes, I say *destructive agencies!* We must face the facts. Can we, claiming to be intelligent practitioners, say, when in nine-tenths of the mouths that are operated upon for filling and artificial teeth this prevailing disorder is remaining, that it is honest to those we profess to serve? Will these inflamed conditions of tissue, exuding a copious flow of vitiated secretions, teeth and roots incrusted with fœtid concretions; I say will this retard or accelerate a better state of things? Can we save teeth by such neglect? Can we, I ask, continue to tell patients that these deposits protect the teeth from decay? This *is* told the people to-day, and by practitioners who claim much; or that there is no remedial treatment? or that "I can treat those cases as well as any one?" (and such men send out and *borrow* a set of instruments to do it with!) Well, this is human, I know, but it is not divine.

Now, what is duty, in this day of yet uneducated numbers of practitioners in anything like a sufficiency to meet the demand that would be made. If we would tell the patients that we were glad to say their case can be successfully treated, but—I know this, with many, is a hard thing to say—that some one that *has* acquired the ability beyond the ordinary degree ought to take charge of it, would these cases be any worse off by doing so? and would not our patients be glad of such favor to them? This, doubtless, will look a little personal, but I might as well say it as to keep on meaning it. Do we know also that our patients do condemn us for our neglect and deception, when they have it demonstrated to them that there is a remedy for their distress? And it has been not a little mortifying, after they have been misinformed, for them to present their mouths in a good state of health as a contradiction, and I am sorry to say that some have been met with the remark (judge this as you may): "Well, what good will it do? It will all come back and be as bad as ever." Such men are "either weak or wicked;" I hope weak. There is an advanced feature of practice coming on the stage that will demand our intelligent attention; and already with those who have been broad enough to accept demonstrated facts, they have come to recognize another fact, that the healthiest possible condition of roots and their surroundings is an absolute necessity to secure the best results. I am referring to the artificial crown setting and the bridge work, or teeth without plates, a practice that is agitating the profession so hopefully at the present time. The introduction of this advanced practice will, ere long, do more to bring about a helpful state of things for the suffering public than the most sanguine of us could have hoped. What I have seen of it has impressed me with a new spirit, and that the day of extraction of valuable teeth, as a practice, has reached its ultimatum, that the premature distortion of the human face will not much longer be tolerated by the intelligent people, and that disgusting plates, in their best estate, are to be laid away to a large extent, and give place to a more useful, cleanly, and healthy condition of things, intelligent practice is proving in hundreds of cases, and it is utterly useless to shut the eyes to what no one of judgment can deny after an unprejudiced inspection. When I say that I have seen hundreds of cases in various conditions of circumstances, from ten years' use to the latest date, admitting some failures growing out of avaricious desires and utter lack of principle, and because of ignorance too much confided in—which are all too common weaknesses—I say, setting all these aside, I am compelled to say from a confirmed knowledge that this must become an acknowledged advanced practice. I say again, as I have already by letter to a friend, after seeing—must I say the base? no, I will say the weak, puerile, mistaken misrepresentation of facts given out to the profession at large, published in the July number of the Cosmos; such statements, "if the entire opposite state of things does not exist, and prove what I have said, so much in common with others, of the value of a clean and healthy oral cavity, as a general practice, in a sense that my most ardent desires could hardly have hoped to have seen (for what I have said), call me a fool, an imbecile." But I am bold to claim that the repute I have established in my profession will give me the credit of some judgment, and some above the ordinary. I say more. And to them who are watching for the words of those who *cannot*, nor *would not*, for their lives, mislead them, I say this, and I do it with sorrow. In all my acquaintance of my profession, I have never seen such a pitiful misstatement of *known facts* as the published one against the "much vaunted (so-called) bridge work." This is not my verdict alone, but it is being daily said by many intelligent practitioners—yet by some in a whisper—a "*great mistake.*" And, I add my own voice, as if it were my last testament, to the same declaration—it is a great mistake—a defamation of a valuable improvement. Time, in the near future, will also proclaim and prove it to be true.

[Gentlemen, you will all sustain me when I say that local controversies, occasioning bitter prejudices and unfriendly relations, should have no part in fixing our judgments of the value of things and methods of practice.] We no longer hang men, or burn them at the stake, for their opinions, but the more modern method is "sitting down on them." Any man that has a conscious daily sense of doing his duty, although there may be an effort to cast obloquy upon him because of some unseen motive or purpose, need have no fear, for ignorant prejudice can be no match for unselfish performance of duty.

Now, I imagine that some one is saying that I am making an omnibus of this paper. Well, if any think so, bear with me until I am done with the subject. The possibilities of this bridge work in connection with loosened teeth have been so favorably demonstrated to me that I would not be faithful to my subject to withhold the facts in my possession. I will give a sample case. In the mouth of a lady of about 45 years of age, eleven beautiful teeth had become very much loosened, and, by an extensive waste of both soft and hard tissues, so much leverage had been produced that it was becoming a question of short time only that they could be kept in their place. They were doomed as they were. The idea was suggested that these teeth be put into a bridge and be connected with the firmer teeth. This was done by crowning each of them and forming a bridge connection, putting them virtually into a straight-jacket. They had been in use, when I last saw them, about two years. It is a success, so far as practical utility and health are concerned. This is an extreme case, but what proof of the value in general practice. Do not understand me as presenting this testimony as a limit of my observations. Gentlemen, if you desire liberality of thought, you must travel liberally, and not in the path of authority alone. If you have not tried it, try a deviation occasionally among the so-called "commonalty," even at the expense of what may seem some adverse criticism. Criticism will always serve as a tonic to a true man. Retrospect is a valuable employment to one who is looking for what is of value, for from it we are able to gather what has been gained of good out of experience.

It will be very apparent, from what I have said, that I have improved the opportunity to declare myself emphatically, and I feel I am warranted in doing so. You know I have done some traveling, and possibly some may have thought that I have gone into some unpopular places; and such may have said, if I had been he I would have kept clear of these tabooed localities. Yes, but you would not have had the experience, nor be able to deduce the valuable *facts* gained. I now know both sides of the question, and I know more. I have an audience outside of this organization, scattered up and down through the earth, and these practitioners are, many of them, thirsting for the unprejudiced truth, and I propose to be faithful to them. No words of mine shall knowingly mislead them. One thing of great value I have had verified by my peregrinations, and that is the undeniable proof of what has been said regarding the success of the Riggs' treatment over the unrecognized part of the Riggs' feature, viz: the non-nutrient condition of the edge of the process. Not a few cases have been shown me by practitioners that claim knowledge, but deny the feature named, and thus ignore the necessity of giving it attention. This point was vigorously discussed by us in the presence of other practitioners, and when they were compelled to acknowledge the intelligence and proof, dodged the issue by saying: Well, after all, it is *business;* you can give them lighter and less hurtful treatment, and send them off happier, and they do not know the difference, and we get more money out of them in the end. Yes, and the people who intrust them with their teeth are deceived by being told that they are getting the legitimate treatment for less money and less hurt.

Regarding this part of my paper, should I seem a good deal in earnest, I trust you will consider that special concentration of thought, purpose, and practice necessarily must generate a greater amount of interest than any generalizing will do. While in a general way it is acknowledged by all practitioners that the presence of deposits is prolific in its manifestations, and that it is quite necessary to do something for the removal of them, yet I am compelled to say that this only applies in a very superficial manner. Men say, "remove tartar; why, any one ought to do that; who does not?" Now, I must further say, so long as the prevailing thought is that "tartar" is the cause of this general and destructive malady, I feel that just so long will the mass of men apply such a superficial view of the treatment. But as it comes to be understood that "tartar" is a result of a systemic disorder, and when it is further understood that there is vastly much more associated with it, which cannot be grasped by simply a hard, mechanical act, then I think we will begin to see a more hopeful sign of increased helpful practice, that shall prove alleviative in a much larger sense.

Of no one thing am I more convinced than of the necessity of the intelligent consideration of the feature first brought to our notice by Dr. Riggs, and that is the non-nourished condition of the edge of the alveolar process, and which is manifested in such a multiplicity of cases. Case after case has come to my notice where the removal of deposits has received attention, and, of course, an improvement was shown by a bettered condition of health. By a revision of such cases, attention to this disturbed portion of the process has proven the efficacy of such treatment by showing a decided improvement over the first. I do not hesitate to say that this point is outside of controversy; it is a settled question to the intelligence of a comprehensive mind. And it is within the field of demonstrative practice, that is, is being constantly demonstrated. So far as my own experience has shown, I see no reason for much change of form of instruments for making these operations as devised by Dr. Riggs. A great variety of forms have invited my attention, but I do not find the mass of them of much value, beyond doing *just short* of the thing *needed* to be done. There are five instruments of the set devised by Dr. Atkinson that have engaged my attention more than any others, and I have combined them with the Riggs set. The S. S. White Co. have produced for me a beautiful set of these, which I call my combination set, making in all eleven instruments. They are furnished with beautiful white ivory handles, all of the form of handle given by Dr. Riggs, and to be used by the hand and not by the mallet, as designed by Dr. Atkinson.

There are occurring not infrequently places where the circular edged instrument, like the form of Dr. Atkinson's, proves very efficient by a push movement instead of the rotary motion of the Riggs instrument. If the criticism is still in the minds of men that the Riggs instruments are ponderous, formidable, etc., all the reply I can make is, that in my hands nothing could be more delicately and dexterously manipulated, nor, I think, with better results, the best that can be obtained. I do not yet see indications sufficient to impress me that this field of practice is being cultivated to any extent, certainly paramount with its importance. So far as the practice that comes into my hands, sent to me by practitioners both dental and medical, I find it encouraging, but as I come in contact with the mass of practice and direct attention to the necessity of the service I can render, I am met by the almost stereotyped reply: "Why, I have had the tartar removed repeatedly." And yet I find deposits that are all the way from ten to twenty-five years standing, the soft tissues about the teeth badly congested, hemorrhagic by the least disturbance with brush and mastication; also a copious discharge of viscid secretions, making, in a large number of instances, a disgusting and filthy condition, accompanied with an odor that, if the patients could realize it, would make them hasty for the remedial service that has been applied in hundreds of cases. I am constantly asked by dentists (and frequently by patients, because they receive this information from dentists): After your treatment, and such good results as are claimed, will not all this trouble return? I answer most emphatically, no, and in a small degree if at all. If the case has been one that has progressed to an advanced condition and is associated with a hard, vitreous deposit, this indicates that *time* has been a factor in the accumulation, and it being properly removed—even should it be neglected—it would require an extended period for it to present the same or similar conditions.

Again, there are not a few cases that *do not* exhibit deposits in connection with the disorder, but which manifest an advanced stage of destruction of both soft and hard tissues. I answer regarding such that the effect of the treatment produces such a radical change, not only local but constitutional, that the increase of physical vigor fortifies the system against any rapid return of the difficulty. And let me say more; you must bear in mind that *special interest* in these cases makes an impression of a greater or less degree on the mind of the patients, and they become partners in the future care of the mouth, which secures against the retrograde process.

I am asked also, will patients submit to the operation readily, and acquiesce in the continued operations necessary for the best results. I have no difficulty in that respect. I study the character of my case and my patient, and do by them as I do in all my practice, seek to establish relations of confidence; and I operate the length of time that my judgment indicates will bring about the most success with the least discomfort. This being done in *general practice* takes away a vast amount if not the whole of the "bugbear" of practice. Do you find an appreciation that brings encouragement of remuneration financially? Let me give you an extract from a letter lately received from a prominent American dentist now in practice in Europe. He writes me thus: "Doctor, the special work that you are so much interested in *will*, in the next *fifty years*, become so intelligently impressed upon the minds of the people by a constantly demonstrated practice that they will then *demand* this surgery and will cheerfully remunerate for such services, *more readily* than they do now for the filling of holes in teeth." My hope is greater than his. My experience has been that all the cases put into my hands by special direction of dentists and physicians have proved very amicable indeed; such patients come prepared for special service and to pay my fee. I can say, and with pleasure, that not in a single case have I had anything but the pleasantest relations. But where I meet cases in general practice it is quite another thing. The majority are not aware of the true condition of their mouths, and correspondingly few are in any sense prepared to readily accept the *facts*, even when moderately stated, yet I am in no sense ever thwarted in my determined purpose to do all in my power to perpetuate this special feature of practice by my words, and earnest ones if necessary, as occasion may require it, and constantly associated with an intelligently demonstrated practice. By this I can reasonably hope that the way shall be made easier for those who may follow me. I can only hope that the time will come when the schools, out of which we have reason to hope the better part of the coming profession shall be prepared for their work, rather than graduated by State societies, shall evince a more tangible interest in the intelligent teaching and clinical demonstration systematically and continuously placed before the students by some one qualified. Until this is done I am *sure* that anythinglike the proportion of those in need of this service will be compelled to bear the affliction so tenaciously fastened upon them.

I am aware that I shall be criticised, as I have been many times, for what I say regarding the wholesale neglect that I charge upon the profession. Yet I am fully able to prove what may seem to be extreme remarks. Not long ago I published some statements in the Missouri *Dental Journal* regarding the large number of cases that had come under my examination during a certain length of time, and it came to my knowledge that I was harshly judged for such so-called "untruthful statistics;" but I know the *better* knowledge sustains me in all I have said, for I have numerous written and oral evidences of this. To say that this important subject has the tithe of a hold and interest upon the general practice cannot be sustained by the facts; but I recognize this that it is for a want of intelligent understanding to some extent, and to a much larger extent a want of interest. Yet progress is the watchword, and we have but to take a retrospect of the past to derive encouragement for future time.

Gentlemen, the public are becoming more and more enlightened. Be not discouraged or deceived. Truthful, intelligent practice *will* assert itself; the lever of faithfulness cannot be destroyed. I wish to leave this paper in the minds of the profession, and to help them to intelligently digest it I will refer them to an editorial in the 22nd volume of the Cosmos, page 207. In this I think they will find much helpful aid in analyzing not only this paper but those on all other subjects.

I think, also, that not only the readers of journals will find much help by carrying out the suggestions of this article referred to, but also the editors of journals themselves, if they are willing to accept its teachings.

G. A. MILLS.

Read at a meeting of the New England Dental Association, Providence, R. I., October 4, 1883.

